# Supplementary material for: Modelling continual learning in humans with Hebbian context gating and exponentially decaying task signals
Source: PLoS Comput Biol. 2023 Jan 19;19(1):e1010808. doi: 10.1371/journal.pcbi.1010808 (PMC9851563; doi:10.1371/journal.pcbi.1010808)
Supplement: S1 Methods — (DOCX) [file pcbi.1010808.s001.docx]

**Supplementary Methods**

**Neural network simulations with trees stimuli**

To verify that our approach could be extended to slightly more complex input datasets and architectures, we repeated the experiments with MLPs with two hidden layers that were trained on a down-sampled version of the fractal tree images from the original paper.

**Stimulus Design**

Stimuli were images of fractal trees that varied in five discrete steps in terms of their density of branches (“branchiness”) and leaves (“leafiness”) and were pasted onto a grey background. We took the dataset of 50000 training and 10000 test images that was used in Flesch et al., 2018 and down sampled each image to 24x24x3 pixels. Pixel values were encoded as floats in the range from 0 to 1.

**Neural Network Architecture**

The neural network was similar to the ones described in the main text. The input layer consisted of 24*24*3=1728 units that received the flattened RGB images, and two additional task units which received a one-hot encoded context signal. Inputs were passed through two hidden layers with 100 ReLU non-linearities each. The output was a single node with sigmoid non-linearity.

**Training Procedure**

All training procedures were similar to those described in the main text. We used the same weight initialisation and performed a hyperparameter search over a range of values for the SGD and Hebb-update learning rates, as well as a “context offset” parameter that was multiplied with the one-hot encoded context signal. The purpose of this scaling was to get a high enough activation from the context units, relative to the input units. The baseline network was trained with a learning rate of $\epsilon= 0.001854$and context offset of of $c=1$ for interleaved and a learning rate of $\epsilon= 0.001968$and context offset of $c=4$ for the blocked curriculum. The Hebbian network was trained with earning rates $\epsilon=0.001968$ and $\eta=0.000849$ and context offset of $c =4$. For comparisons with human data, we trained the sluggish Hebbian network with sluggishness values ranging from $\alpha=0.05$ to $\alpha=1.00$ in 30 steps and learning rates $\epsilon=0.001968$ and $\eta=0.000849$ and chose the sluggishness value that minimised the difference between choices made by humans and the neural network outputs. For each network, we collected 50 training runs with independent random weight initialisations. To stabilise training of the Hebbian network, we had to restrict Hebbian weight updates to the connections from the two context units to the hidden layer.

All networks were trained for 100 episodes (50 training trials per episode, spanning all 5x5x2 combinations of branchiness, leafiness and tasks, but with randomly drawn specific exemplars) and evaluated on all 10000 test stimuli to assess generalisation performance. All reported results (except for learning curves) are based on those test trials.
