# Supplementary figures and images for: Modelling continual learning in humans with Hebbian context gating and exponentially decaying task signals
Source: PLoS Comput Biol. 2023 Jan 19;19(1):e1010808. doi: 10.1371/journal.pcbi.1010808 (PMC9851563; doi:10.1371/journal.pcbi.1010808)

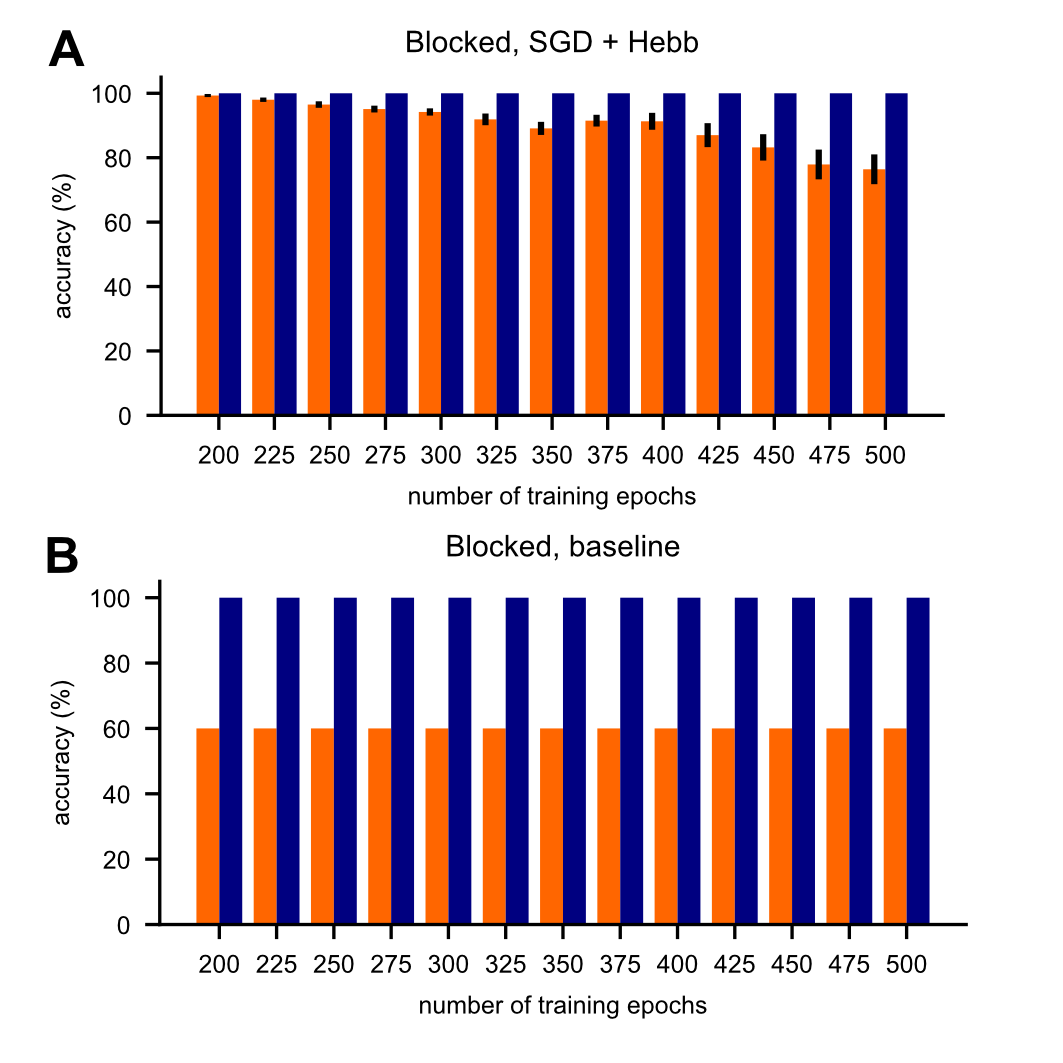

Supplement: S1 Fig — (A) Impact of block length on forgetting of the first task in network trained with SGD and Hebbian updates on blocked curriculum. Hyperparameters were optimised for a block length of 200 epochs and kept the same for all other training lengths. Even with more than twice as many training epochs, the network was still able to perform the first task well. (B) Same as (A) but for network trained without Hebbian updates. (TIFF) [file pcbi.1010808.s002.tiff]

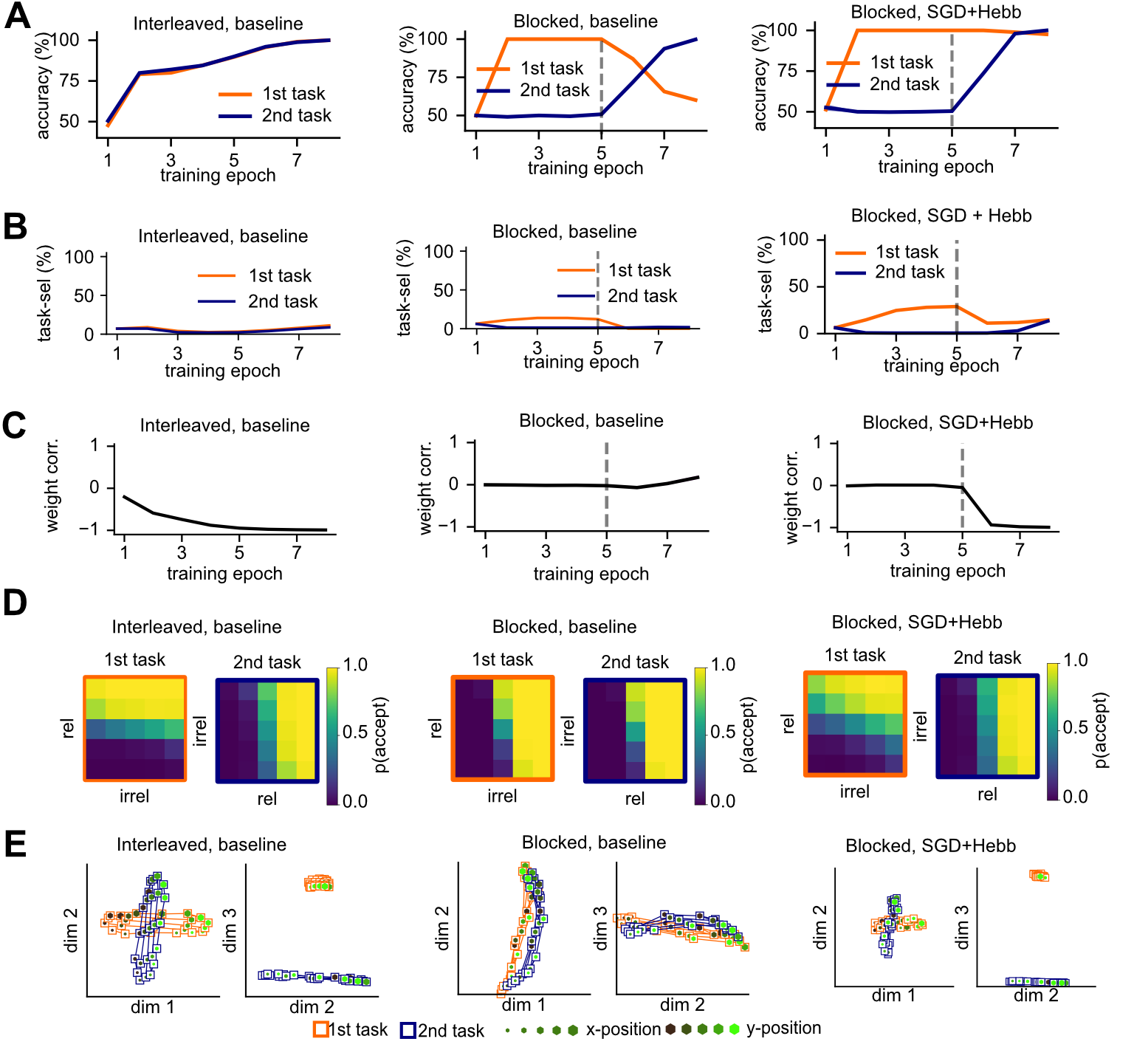

Supplement: S2 Fig — We repeated the main simulations with the two baseline nets, which were only trained with vanilla SGD, either on interleaved or blocked data, and the network trained both with SGD + Hebbian updates on a blocked curriculum. Hyperparameters were optimised for each net separately. The results show that key findings can be replicated, even if the networks receive as few trials as human participants in the original study. (A) Training accuracy for the vanilla net, trained on interleaved or blocked trials, and the Hebbian net, trained on blocked trials. The vanilla net converges on interleaved data but suffers from catastrophic interference under blocked training. In contrast, the Hebbian network’s performance on the first task remains at ceiling. (B) Fraction of units that became purely task selective. While overall, the fractions were much lower than in the networks trained on 200 episodes, more task-selective units were found in the vanilla network trained on interleaved data and the Hebbian network trained on blocked data, compared to the vanilla network that received a blocked training curriculum. (C) Correlation between context weights. Interleaved training with a vanilla network and blocked training with the Hebbian intervention both induced anti-correlated context weights. The vanilla network trained on blocked data failed to utilise the context signal. (D) Network responses. The vanilla network trained on blocked data treated the first task as if it was the second. The other two networks learned accurate estimates of the category boundaries. (E) MDS on the hidden layer activity patterns. The vanilla network trained on blocked data filtered out the dimension that was irrelevant for the second task but applied the same strategy to the first task. In contrast, the vanilla network trained on interleaved data and the Hebbian network trained on blocked data formed orthogonal representations, also consistent with our previous reports. (TIFF) [file pcbi.1010808.s003.tiff]

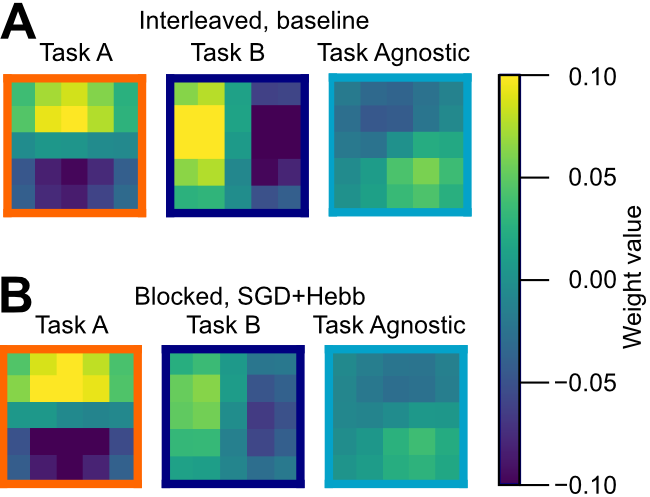

Supplement: S3 Fig — (A) Learned weights for vanilla network, trained on interleaved data. Each heatmap shows averages of the weights from the input layer to hidden units that are selective to either the first or second task, or task-agnostic, reshaped from a 25x1 vector to a 5x5 matrix to resemble the dimensionality of the input images. The plots indicate that task-selective units are associated with weights that select for the task-relevant dimensions (position along the x- and y- axis respectively), while the task-agnostic units code for stimuli that have the same response across tasks (= congruent trials). (B) Same as (A) but for network trained additionally with Hebbian updates on a blocked curriculum. A very similar structure was observed. (TIFF) [file pcbi.1010808.s004.tiff]

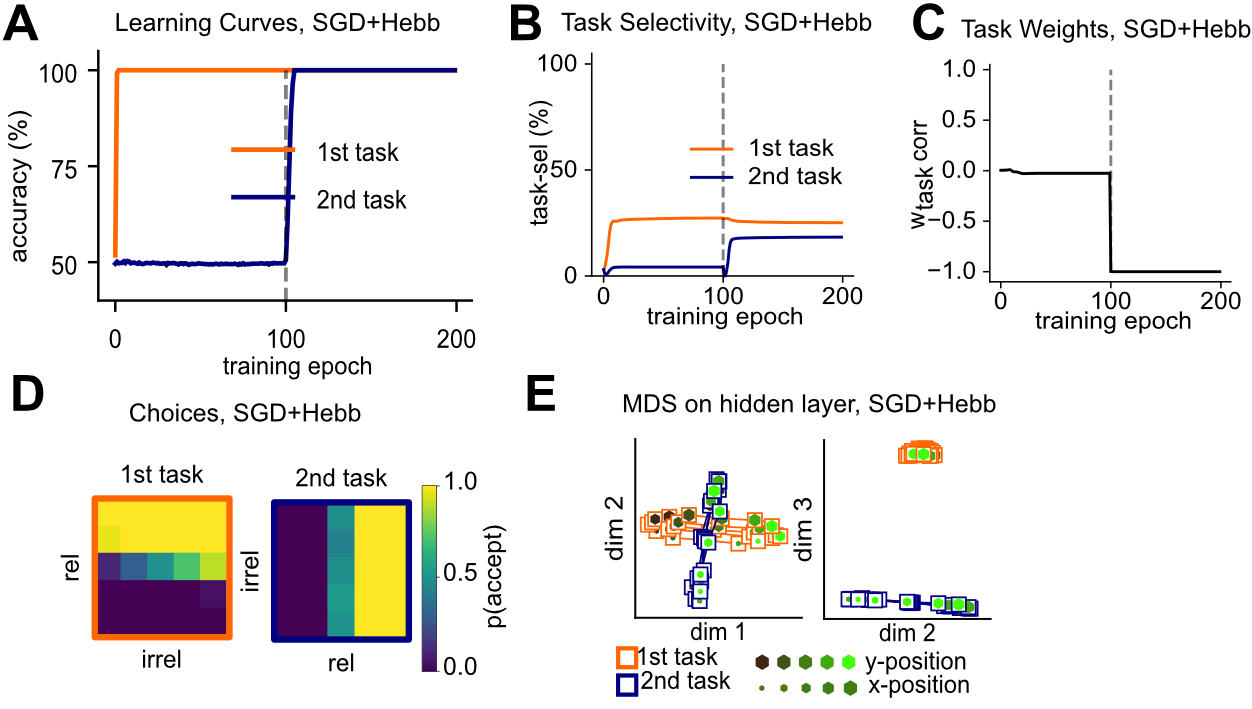

Supplement: S4 Fig — Our approach solves two problems, how to discover the context signal and how to use it to gate out irrelevant dimensions. The former requires that the context/task signal is the largest principal component in the dataset. We note, however, that the mechanism still protects against catastrophic forgetting if the Hebbian updates are only applied to task units (instead of all inputs) and it is assumed that the task signal has already been identified among the inputs. This is shown here. (A) Learning curves for network trained with SGD and Hebbian updates on blocked curriculum. (B) Emergence of task-selective units in the hidden layer. (C) Correlation between weights from task-units in the input layer to the hidden layer. (D) Choices made by the network at the end of training. (E) Projections of learned hidden layer representations into three dimensions. (TIFF) [file pcbi.1010808.s005.tiff]

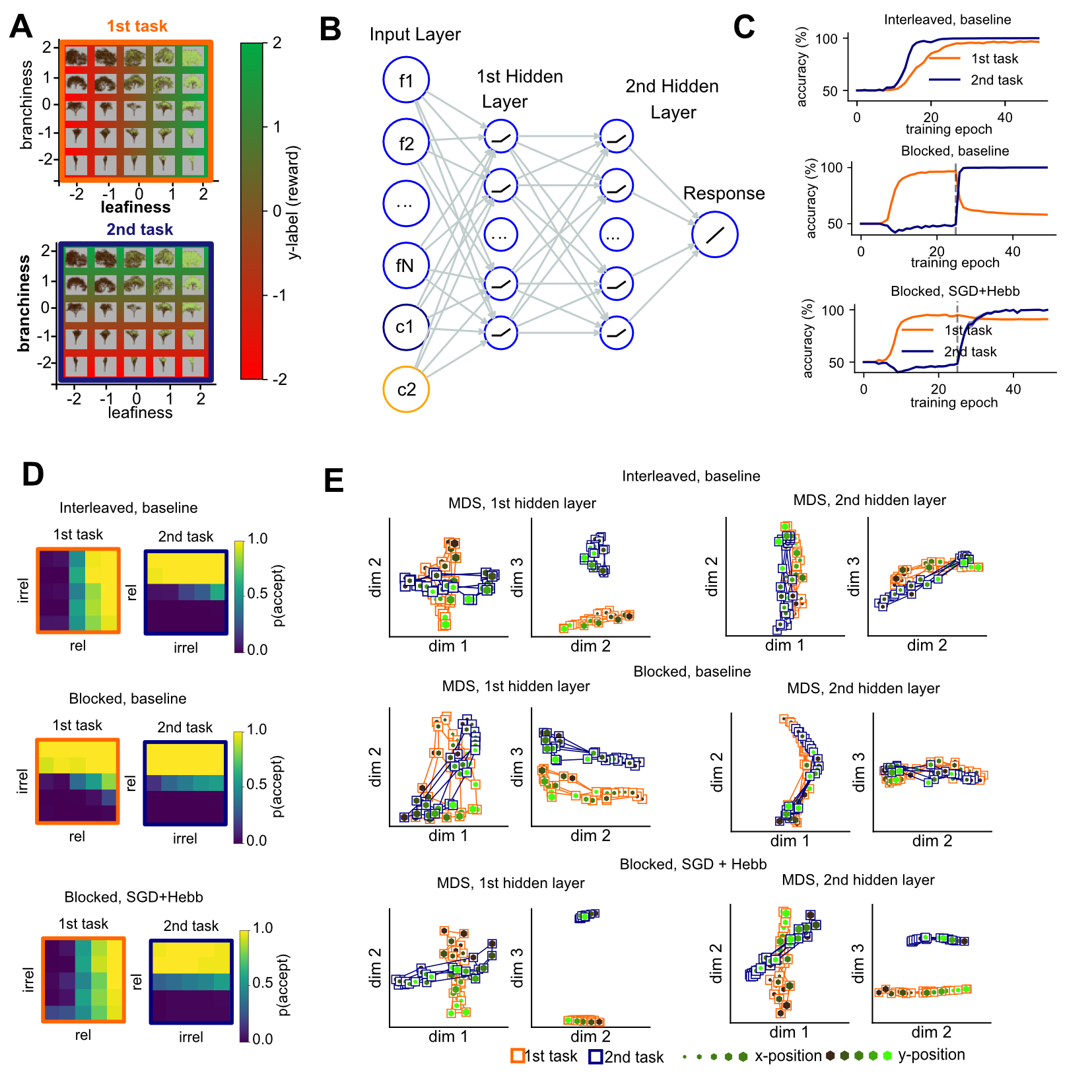

Supplement: S5 Fig — (A) Stimuli and task rules. Instead of Gaussian blobs, we used RGB images of fractal tree images from the original study. Trees varied in five discrete steps in terms of their density of branches (“branchiness”) and leaves (“leafiness”). Only one of the two dimensions was relevant in each context/task. (B) Network architecture. Once again, we used a feed-forward neural network, but this time with two hidden layers with ReLU non-linearities. Inputs were flattened and normalised RGB images of trees, together with a one-hot encoded task signal that indicated whether the network was doing the first or the second task. (C) Learning curves for vanilla network trained just with SGD on either interleaved (top) or blocked (middle) data, and Hebbian network that was trained on blocked data with SGD and Hebbian updates (bottom). Learning curves were similar to those observed with the simpler network and Gaussian blobs. (D) Outputs of the three networks. The vanilla network trained on blocked data treated the first task as if it was the second. The other two networks learned accurate category boundary estimates. (E) MDS applied to patterns in both hidden layers of the three networks. The baseline network, trained on interleaved data formed orthogonal representations in the first hidden layer (1st row, left) and parallel representations in its second layer (1st row, right). These parallel representaitons were obtained by rotating one of the task manifolds from the previous layer by 90 degrees, to bring both into the frame of reference of the response, so that leafiness of the first task was mapped onto the same axis as branchiness of the second task. The network trained on blocked data, in contrast, just represented branchiness, which was relevant for the second task, and did not distinguish between contexts (2nd row). The network trained with Hebbian updates on blocked data (3rd row), in contrast, learned similar representations as the baseline network trained on interleaved da [file pcbi.1010808.s006.tiff]

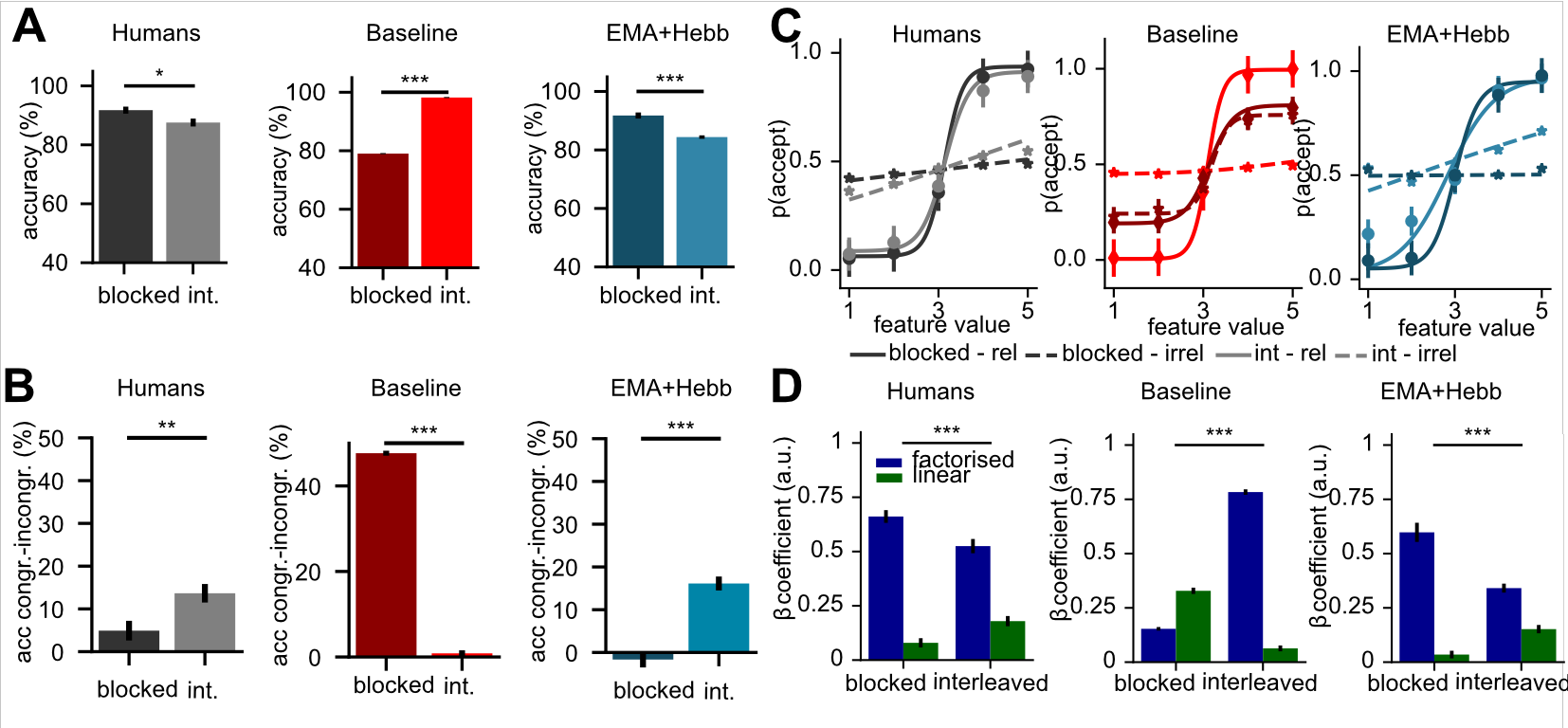

Supplement: S6 Fig — Same analysis as shown in Fig 5 (main text), but for MLPs that were trained on the same fractal tree images as human participants. (A) Test phase accuracy. Both humans and the sluggish network trained with additional Hebbian updates (EMA+Hebb) perform better under blocked compared to interleaved training (humans: T(93) = 2.32, p = 0.022; EMA+Hebb: T(98) = 3.81, p = 0.0005). The baseline network, only trained with SGD (“Baseline”) suffered from catastrophic interference (accuracy blocked < interleaved: T(98) = 73.94, p < 0.0001). (B) Congruency effect. Just like human participants, under interleaved training, our model performs worse on incongruent compared to congruent trials (humans: T(93) = 2.73, p = 0.0075, EMA+Hebb: T(98) = 7.03, p < 0.0001). (C) Sigmoidal fits of choices made by human participants and the two types of MLPs. The EMA+Hebb network makes choices similar to human participants, with more intrusion from irrelevant dimensions under interleaved compared to blocked training (humans: T(93) = 28.25, p < 0.0001, EMA+Hebb: T(98) = 5.13, p < 0.0001). The baseline network doesn’t capture human choice patterns. (D) Fits of the factorised and linear model to human and network choices. For humans and the EMA+Hebb model, the factorised model fits better under blocked than interleaved training (humans: T(93) = 3.07, p = 0.0028, EMA+Hebb: T(98) = 5.14, p < 0.0001), while the opposite was true for the linear model (humans: T(93) = -3.12, p = 0.0024, EMA+Hebb: T(98) = -4.39, p = 0.0001). Error patterns of the baseline model show the opposite pattern (Factorised model blocked < interleaved: T(98) = -40.86, p < 0.0001, linear model blocked > interleaved: T(98) = 13.06, p < 0.0001). (TIFF) [file pcbi.1010808.s007.tiff]

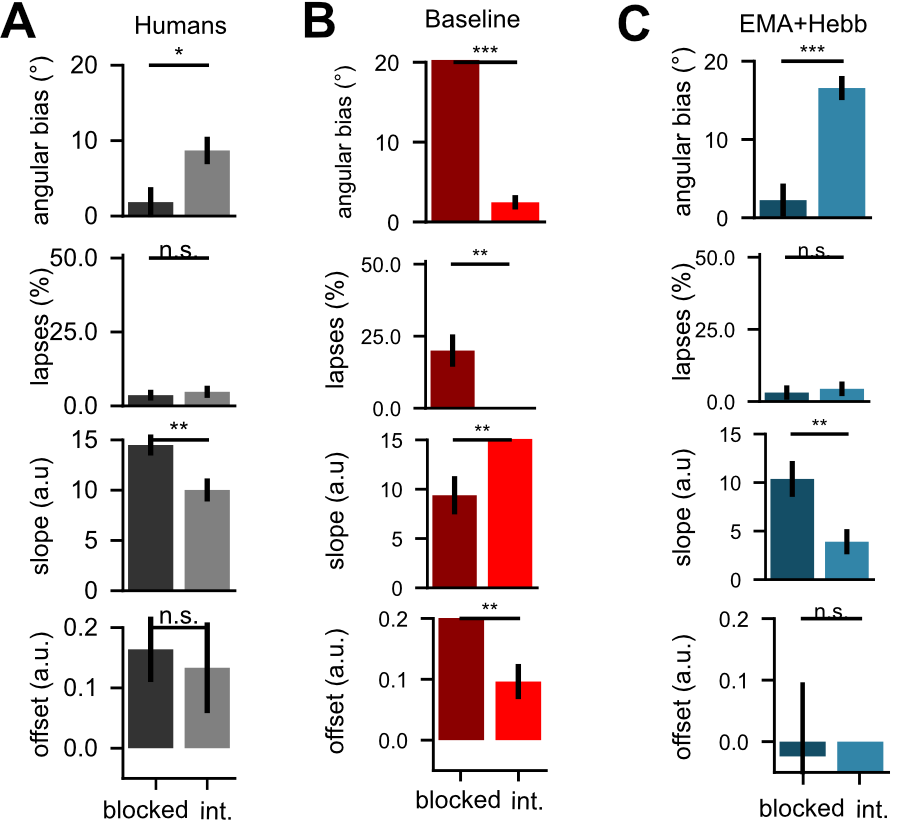

Supplement: S7 Fig — (A) Fits of a psychometric model with four parameters to choices made by human participants, decomposing error patterns into (i) angular bias of category boundary (ii) lapse rate (iii) slope and (iv) offset of sigmoidal transducer. Both the angular bias and slope differed significantly between participants trained on blocked and interleaved curricula (bias: T(93) = -2.54, p = 0.0127, slope: T(93) = 2.88, p = 0.0049). (B) Same as (A) but fitted to the MLP that was trained on fractal trees images with vanilla SGD. (C) Same as (A), but fitted to the outputs of an MLP that was trained on the trees images with sluggishness and additional Hebbian updates. Patterns resembled those observed in human participants (bias: T(98) = -5.47, p < 0.0001, slope: T(98) = 2.86, p < 0.0001). (TIFF) [file pcbi.1010808.s008.tiff]
